# A New Method of Extracting Temporary Teeth

**Published:** 1874-08

**Authors:** 


### EDITORIAL. ETC.

*A New Method of Extracting Temporary Teeth.*—The editor of the *Missouri Dental Journal* is responsible for the following:

"Among the many useful little articles which it is always convenient to have at hand in a dental office, is small rubber tubing, in sizes from an eighth to a fourth of an inch. The uses to which it is adapted, viz:—as a means of separating teeth, holding the rubber dam on the molars or other teeth when central cavities are to be filled; as a dam, in connection with the napkin or bibulous paper when the Barnum dam is not at hand. In correcting irregularities of the dental arch its use in various ways has suggested itself to the intelligent dentist. For this purpose it is an indispensable article with us; in fact we feel that we might say, without fear of successful contradiction, that any irregularity of the arch, no matter how great, can be corrected by a proper use and application of these little rubber rings and the silk ligature. But we have now to record a new use for this useful little article, namely, the extraction of the deciduous teeth. Some of our readers may perhaps know from sad experience the effect of leaving a rubber ring for a day or two surrounding the neck of a tooth. If it was an incisor or canine, you had the mortification of seeing your patient return with a very sore tooth, which was gradually being drawn from its socket. We have had a little experience of this kind, and it has taught us useful lessons. We have learned from it never to leave, for a moment, a rubber ring on a tooth which we did not desire to extract, without having a ligature passed through it to remind us of its presence. And again, if we desired to extract a deciduous molar for a timid child the rubber ring furnished the most convenient and ready means of doing it without pain, and to the great surprise and gratification of our little patient. All we found to be necessary in the case was to slip one of the rings over the tooth, force it gently under the gum and dismiss our patient with the injunction not to remove it. The tooth would gradually loosen and finally fall out, the rubber ring having surely, silently, and painlessly done the work of the dreaded forceps."

The above reminds us of a case which occurred in the practice of a dental practitioner of this city some years ago, where a valuable tooth was destroyed by a band of rubber, which had been adjusted to its neck for the purpose of arresting the mucous secretion of the gums during the operation of filling an approximal surface cavity, being permitted to remain through carelessness, after the patient was dismissed. When the tooth was removed, which could have been done with the fingers, so loose was it, the rubber band was found tightly embracing the apex of the root, having gradually made its way to this point from the neck of the tooth.

